# Photoswitchable Silver(I) Complex with Anticancer and Antimicrobial Potential

**DOI:** 10.1021/acsomega.6c00430

**Published:** 2026-03-29

**Authors:** Aleksandra Kręcigłowa, Marta Stolarek-Sipior, Patrycja Jagielska, Kamil Kamiński, Magdalena Skóra, Marlena Gryl, Artur Sikorski, Janusz Rak, Maria Nowakowska, Krzysztof Szczubiałka

**Affiliations:** † Faculty of Chemistry, 37799 Jagiellonian University, Gronostajowa 2, Cracow 30-387, Poland; ‡ Doctoral School of Exact and Natural Sciences, Jagiellonian University, Łojasiewicza 11, Cracow 30-348, Poland; § Department of Infection Control and Mycology, Jagiellonian University Medical College, Czysta 18, Cracow 31-121, Poland; ∥ Faculty of Chemistry, 49646 University of Gdansk, Wita Stwosza 63, Gdansk 80-308, Poland

## Abstract

A photoswitchable silver­(I) complex incorporating two arylazopyrazole ligands was synthesized and characterized. The system undergoes efficient and reversible *trans–cis* and *cis–trans* photoisomerization upon irradiation at 365 and 530 nm, respectively, with the metastable *cis* form exhibiting a thermal half-life of 13 days at 37 °C, indicating potential suitability for photopharmacological applications. The complex showed significant dose-dependent toxicity in both cancer (4T1) and normal (NMuMG) murine mammary gland cells. However, the concentration window around 18 μM was identified in which the *cis* photoisomer is not toxic in both normal and cancer mammary gland cells, while the *trans* photoisomer is toxic in cancer cells and nontoxic in normal cells. Favorably, in cancer 4T1 cells, the *trans* photoisomer was more toxic than cisplatin, which in turn was more toxic than the *cis* photoisomer. Both photoisomers were nontoxic in human prostate cancer (PC3) and nonsmall cell lung cancer (A549) cells. The complex showed higher fungistatic than bacteriostatic activity, with no differences in toxicity between photoisomers. In human keratinocytes (HaCaT), the *cis* photoisomer was nontoxic, while the LC50 of the *trans* one in these cells was 10 times higher than its MIC against *Aspergillus fumigatus*, revealing the potential of the complex as an antifungal agent.

## Graphical abstract

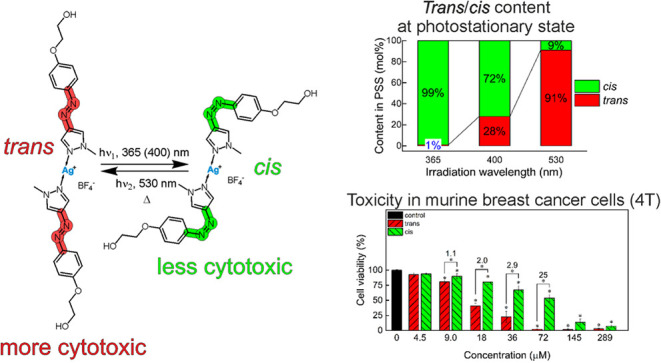

## Introduction

Photopharmacology is a comparatively new branch of pharmacology aiming at achieving control over drug activity using light.^1–3^ Controlling drug activity with light is particularly attractive for a few reasons. Light can be delivered with high spatial and temporal precision, thereby increasing selectivity of an administered drug (a photopharmaceutical) and reducing its adverse effects, which is particularly important in the case of highly cytotoxic anticancer agents. Light, especially from the visible region, is much less invasive than other external stimuli. Moreover, photopharmaceuticals may also respond to other stimuli, e.g., temperature or pH, offering dual stimulus-responsiveness for even more precise therapeutic targeting.^4^

The compounds used to impart photocontrollability to the drugs are typically classified as photoswitches (PS), whose molecules can change shape under light and reversibly activate/deactivate the drug, and photocages, which undergo irreversible photodissociation, releasing the biologically active molecule.^5^ Reversibility of the control provided by the photoswitches may be achieved by photoisomerization (e.g., *trans–cis* photoisomerization of the azo compounds), resulting in the significant change in the molecule structure/geometry. Among azo photoswitches of particular interest are arylazopyrazoles, offering high conversion yields for photoisomerization in both directions and long half-lives of the metastable *cis* photoisomer.^6^ Photoswitches and photocages were successfully applied to change the physiological activity of various types of drugs, such as antibiotics,^7^ as well as endogenous substances, like hormones,^8^ lipids,^9^ and proteins,^10^ including enzymes.^11^

Metal complexes, still underrepresented among photopharmaceuticals, play a crucial role in modern medicine,^12,13^ with applications ranging from oncology^14,15^ to the treatment of infectious diseases^16^ and diagnostic techniques such as medical imaging.^17,18^ Their broad biological utility stems from the unique physicochemical properties of metal ions, especially their ability to form stable and selective coordination bonds with key biomolecules such as proteins, DNA, and enzymes, affecting their essential functions.^19^

While platinum-based drugs (such as cisplatin) are the most widely known and clinically used metallodrugs in oncology,^14,15^ numerous other metal complexes—including those of ruthenium, titanium, gallium, iron, cobalt, gold, and zinc—have also demonstrated significant anticancer activity.^20^ In parallel, a growing body of evidence supports the antimicrobial potential of metal coordination compounds. Of particular interest are complexes of pyrazole derivatives with copper and iron, which have shown growth-inhibitory effects against selected bacterial strains.^21^ Furthermore, complexes of nickel­(II) and cobalt­(III) with pyrazole ligands have been reported to exhibit antifungal activity.^22^

Among the metal complexes of greatest biomedical interest, those of silver­(I) with pyrazole derivatives have attracted considerable attention due to their anticancer potential and broad-spectrum antibacterial and antifungal activity. Gandin and colleagues reported the cytotoxic effects of these complexes against cancer cells, highlighting their potential in chemotherapeutic development,^23^ while Hu et al. demonstrated that silver-pyrazole complexes exhibit strong antimicrobial activity.^24^

The mechanism of silver ions’ action is multifactorial and includes induction of mitochondrial dysfunction,^25,26^ cell wall disruption,^27^ interaction with nucleic acids,^28^ and the generation of reactive oxygen species (ROS).^29^ Silver is also known to interfere with thiol-based redox reactions, which may result in the inhibition of electron transport, impairment of cellular respiration, and inactivation of essential enzymes.^30^ Moreover, silver ions can bind directly to DNA via sulfide bond formation, contributing to genomic instability. These mechanisms are thought to underlie both the anticancer and antimicrobial properties of the silver-based compounds.

The anticancer effects of silver complexes resemble those of platinum-based agents, involving mitochondria-mediated apoptosis triggered by direct interaction with DNA.^31^ Importantly, the biological activity of silver complexes is closely tied to their physicochemical properties, including aqueous solubility, chemical stability, lipophilicity, redox potential, and the kinetics of silver ion release.^32^

Recent advances have also introduced a new class of silver-based antifungal agents, developed by Supuran et al., which target phosphomannose isomerase, an essential enzyme in fungal and yeast cell wall biosynthesis. This enzyme has been shown to contain at least two metal-binding sites. Their interaction with Ag^+^ and Zn^2+^ impairs enzymatic function and compromises fungal cell wall integrity.^33^

In this study, we present another silver-pyrazole-type complex incorporating a photoswitchable arylazopyrazole-based ligand. The *trans–cis* photoisomerization of the ligands is expected to markedly alter the complex’s size and shape from a near-linear geometry in the *trans* form to a more compact one in the *cis* form. We therefore hypothesized that these structurally distinct isomers would exhibit different toxicities. Accordingly, irradiation that drives reversible *trans*↔*cis* interconversion should enable light-controlled, bidirectional modulation of the complex’s toxicity. This idea has already been verified—we previously used this ligand to successfully gain photocontrol over the cytotoxicity of a platinum complex, a cisplatin analog, which showed photoswitchable cytotoxicity across various cell lines, both cancer and normal ones.^34^ We also used the same photoswitch to achieve photocontrol of some biological properties of heparin.^35^ Given the well-documented biological properties of silver complexes, the newly obtained photosensitive coordination compound was assessed for its anticancer and antimicrobial activity, with positive results. The crystal structure of the complex, its ligand, and the intermolecular interactions were also analyzed.

## Results and Discussion

### Synthesis and Characterization of the Photoswitchable Silver­(I) Complex (Ag­(*trans-*PS1)_2_BF_4_)

We used the arylazopyrazole-based photoswitch (*trans-*PS1), reported in our previous publications,^34,35^ to synthesize a photoswitchable silver­(I) complex (Ag­(*trans-*PS1)_2_^+^BF_4_^–^). The synthesis of the complex was based on a modified literature procedure for the synthesis of pyrazole derivatives with Ag­(I).^36^ The Ag­(I) complex was expected to contain two *trans-*PS1 ligands, with pyrazole nitrogen atoms forming two colinear coordination bonds to the central Ag^+^ ion and the tetrafluoroborate anion as the counterion. The complex structure (Figure 1) was confirmed by using elemental analysis (EA) (Table S1), ^1^H NMR (Figure S1), ATR-FTIR (Figure S2), and SCXRD (Figure 2) measurements.

**Figure 1:**
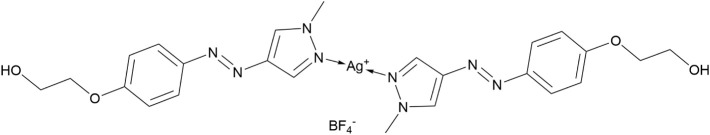
Structure of the Ag­(*trans*-PS1)_2_BF_4_.

**Figure 2:**
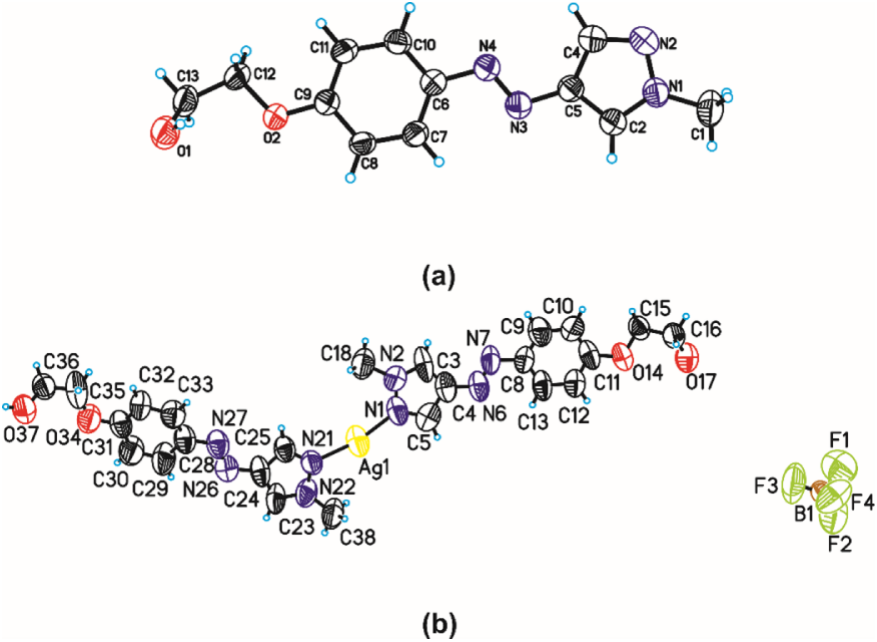
Asymmetric unit of PS1 (a) and Ag­(*trans*-PS1)_2_BF_4_ (b) showing the atom-labeling scheme. Displacement ellipsoids are drawn at the 50% probability level, and H atoms are shown as small circles of arbitrary radius.

The results of the EA of the product were consistent with the assumed elemental composition of Ag­(*trans*-PS1)_2_BF_4_ and clearly different from the theoretical elemental composition of *trans-*PS1 (Table S1). Comparison of the ^1^H NMR spectra of Ag­(*trans*-PS1)_2_BF_4_ and the photoswitch ligand (*trans*-PS1) in DMSO-d_6_ (Figure S1a and b, respectively) indicated that the chemical shifts of their corresponding protons in both spectra are identical. The UV–vis spectrum of the complex featured the main absorption band at 347 nm with the maximum at the same wavelength and minimally wider than the corresponding band in the spectrum of *trans-*PS1 (Figure S2). The ATR IR spectrum of Ag­(*trans-*PS1)_2_BF_4_ (Figure S3a) contained characteristic bands present also in the corresponding spectra of both *trans*-PS1 (Figure S3b) and AgBF_4_ (Figure S3c). The bands characteristic of Ag–N stretching vibrations occur in the far-infrared region (∼400–600 cm^–1^), outside of the accessible wavenumber range of the apparatus. The ultimate confirmation of the assumed structure of the complex was provided by the X-ray diffraction analysis, which indicated that the complex structure is indeed identical to the assumed one (see below).

### Crystal Structure and Intermolecular Interactions

Single-crystal X-ray diffraction analysis reveals that the PS1 ligand crystallizes in the orthorhombic space group *P*bcn, with one molecule in the asymmetric unit (Figure 2a), whereas Ag­(*trans*-PS1)_2_BF_4_ crystallizes in the monoclinic space group *P*2_1_/c, with one Ag^+^ ion, two *trans-*PS1 ligands, and one BF_4_^–^ anion in the asymmetric unit (Figure 2b). Crystal data and structure refinement details are listed in Table S2.

In the crystal structure of PS1, both the pyrazole and benzene rings are nearly coplanar with a dihedral angle of 3.2° and a torsion angle ∠(C5–N3–N4–C6) of −178.3°, whereas the ∠(O1–C13–C12–O2) torsion angle is 68.9°. The molecules in the unit cell are arranged in a herringbone motif (Figure S4). In this structure, there are only weak interactions of C–H···O type connecting dimers and C–H···π between the adjacent dimers. The geometry of weak interactions is presented in Table S3.

In the crystal structure of the complex, slight differences are observed in the geometry of the two *trans-*PS1 ligands: the torsion angles ∠(C4–N6–N7–C8) and ∠(C24–N26–N27–C28) are 178.8° and 175.6°; the ∠(O14–C15–C16–O17) and ∠(O34–C35–C36–O37) angles are 66.1° and 70.2°, whereas the dihedral angles between the pyrazole and the benzene rings are 14.1° and 21.2° (the given values correspond to the *trans-*PS1 ligands with atom numbering N1–O17 and N21–O37, respectively). This observation demonstrates that in metal complexes, both pyrazole and benzene rings of the PS1 ligand do not remain coplanar and show measurable distortions.

In the crystal packing of Ag­(*trans*-PS1)_2_BF_4_, the *trans-*PS1 ligands are connected via O17–H17A···O37 hydrogen bonds, forming chains along the [110] direction (Figure S5, Table S4). These chains are linked by O–H···F (O37–H37A···F2) and C–H···F (C16–H16A···F4, C18–H18B···F3, and C25–H25A···F1) hydrogen bonds, as well as π–π stacking interactions, resulting in the formation of a three-dimensional framework. The geometry of the above-mentioned interactions is presented in Tables S4 and S5.

### Photoisomerization of Ag­(PS1)_2_BF_4_

*Trans*-*cis* and *cis*-*trans* photoisomerizations of Ag­(*trans*-PS1)_2_BF_4_ were studied by using UV–Vis and ^1^H NMR spectroscopies. Electronic absorption spectra of the aqueous Ag­(*trans*-PS1)_2_BF_4_ solution irradiated first with 365 nm and then with 530 nm light are shown in Figure 3a and b, respectively. They suggest that irradiation of the complex with 365 and 530 nm light leads to its *trans–cis* and *cis–trans* photoisomerization, respectively (Figure 4). Irradiation of Ag­(*trans*-PS1)_2_BF_4_ with 365 nm light was accompanied by a decrease in the absorption band at 347 nm and an increase at 280 nm (Figure 3a), which resulted in the formation of a well-defined isosbestic point at 302 nm, suggesting that no side photoreactions took place. Irradiation for a time longer than 45 s did not lead to further changes in the spectra, indicating that a photostationary state (PSS) was established. The ^1^H NMR spectra analysis of the system in which the PSS was reached (see below) indicated that it contained almost exclusively the *cis* photoisomer of the complex (Ag­(*cis*-PS1)_2_BF_4_). Subsequent irradiation of this photoisomer with 530 nm light (Figure 3b) induced *cis–trans* photoisomerization, which was completed within about 740 s. After this time the spectrum of the solution was almost identical to that of Ag­(*trans*-PS1)_2_BF_4_; thus, the *cis–trans* photoisomerization was almost quantitative, and the composition of the solution at the PSS reached was again determined using ^1^H NMR (see below). *Trans–cis* photoisomerization of the complex occurred also during irradiation with 400 nm light (Figure S6a), although it was not quantitative, and, in the PSS, the content of the *trans* photoisomer was about 72 mol % (Figure S6b). Both photoreactions followed first-order kinetics, as expected for the photoisomerizations (Figure S7). Under the applied experimental conditions, the *trans*-*cis* isomerization was about 10 times faster than the *cis*-*trans* photoisomerization (reaction rate constants/half-lives were 0.061 ± 0.004 s^–1^/11 s and 0.00626 ± 0.00005 s^–1^/111 s, respectively; see Figure S7), mainly due to much stronger absorption of the complex at 365 nm than at 530 nm. The shape and size of the complex molecules with both ligands in the *trans* and *cis* configurations differ significantly (Figure 4), and it could be thus expected that both photoisomers may also show distinct biological properties, as indeed was confirmed in the subsequent biological studies (see below).

**Figure 3:**
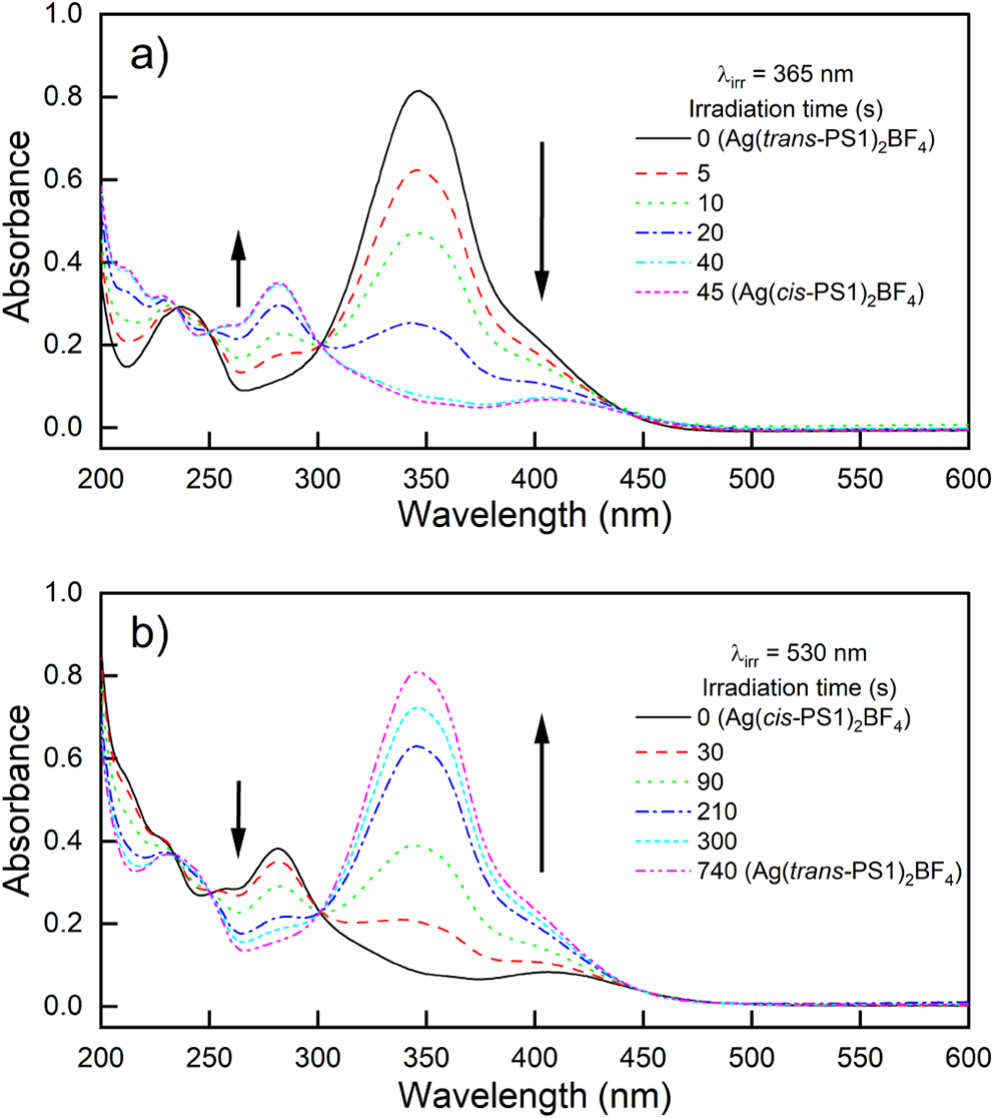
UV–vis spectra of (a) Ag­(*trans*-PS1)_2_BF_4_ and (b) Ag­(*cis*-PS1)_2_BF_4_ irradiated in H_2_O (0.0252 mM) with 365 and 530 nm light, respectively.

**Figure 4:**
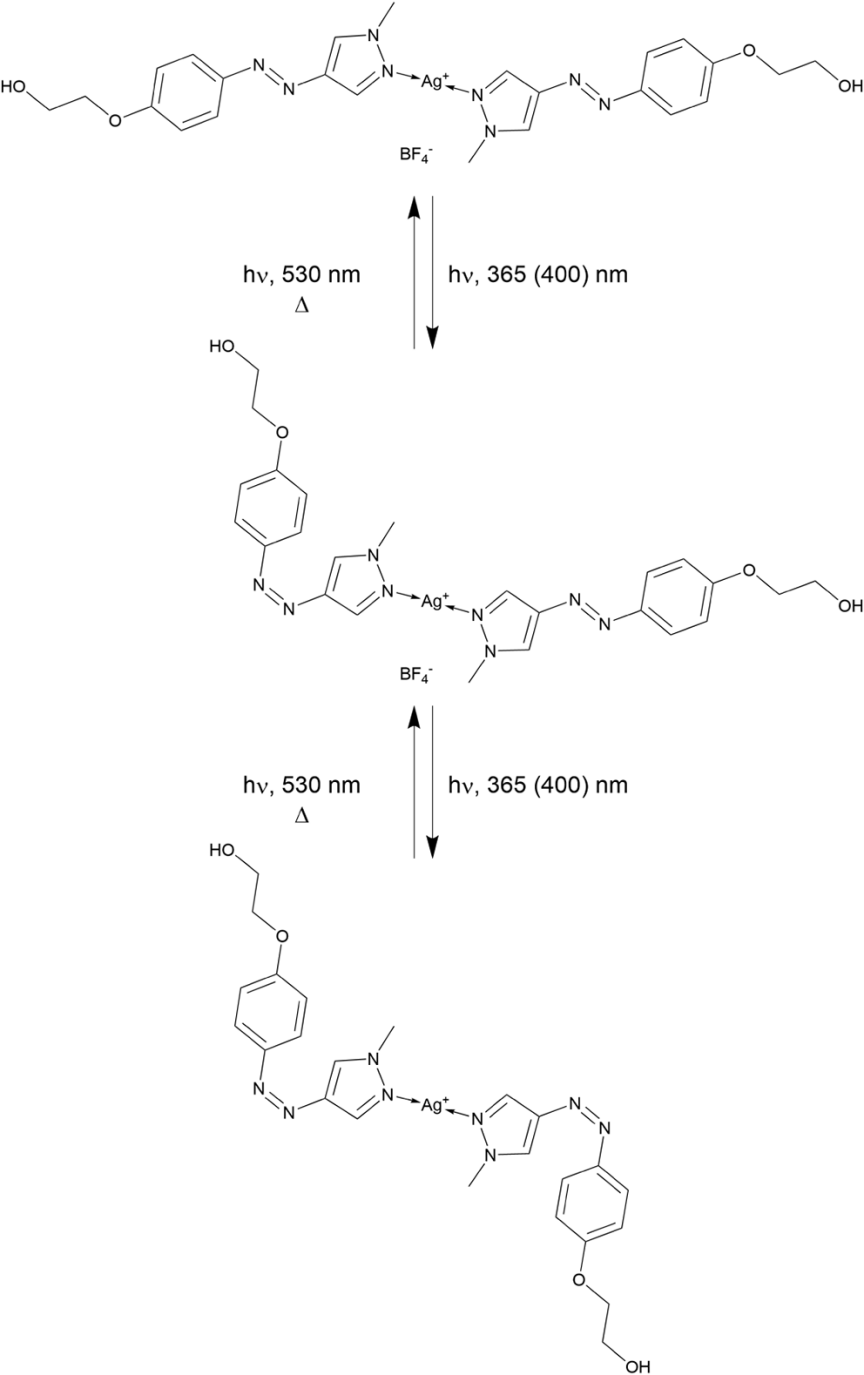
Photoisomerization of the complex.

It should be pointed out that both PS1 ligands in the complex photoisomerize independently; therefore, the formation of an intermediate is expected in which the PS1 ligands have different configurations (Figure 4, middle structure). Isolation of this intermediate product would be highly challenging because of its instability (*cis*-PS1 ligands undergo thermal (dark) *cis–trans* isomerization; see below). Moreover, since its photochemical and biological properties are of secondary importance at this stage of the study, we chose to disregard this aspect in the subsequent investigations.

Quantitatively, the photoisomerization process could be more precisely followed using the ^1^H NMR spectra of the nonirradiated and irradiated complex (Figure 5). Complexation of the silver cation by *trans-*PS1 did not result in the shift of the signals of *trans-*PS1 protons (Figure S1). Similarly, the chemical shifts of the protons in Ag­(*cis*-PS1)_2_BF_4_ (Figure 5) are almost identical to those of *cis*-PS1.^34^ This is in contrast to the analogous complex of *trans-*PS1 with platinum­(II), for which we found a significant shift of the ligand aromatic protons caused by the complexation.^34^ The unchanged chemical shift of the ligand protons after complexation is characteristic of the complexes of the Ag^+^ cation with neutral ligands due to its d^10^ configuration and soft acid character.^37,38^

**Figure 5:**
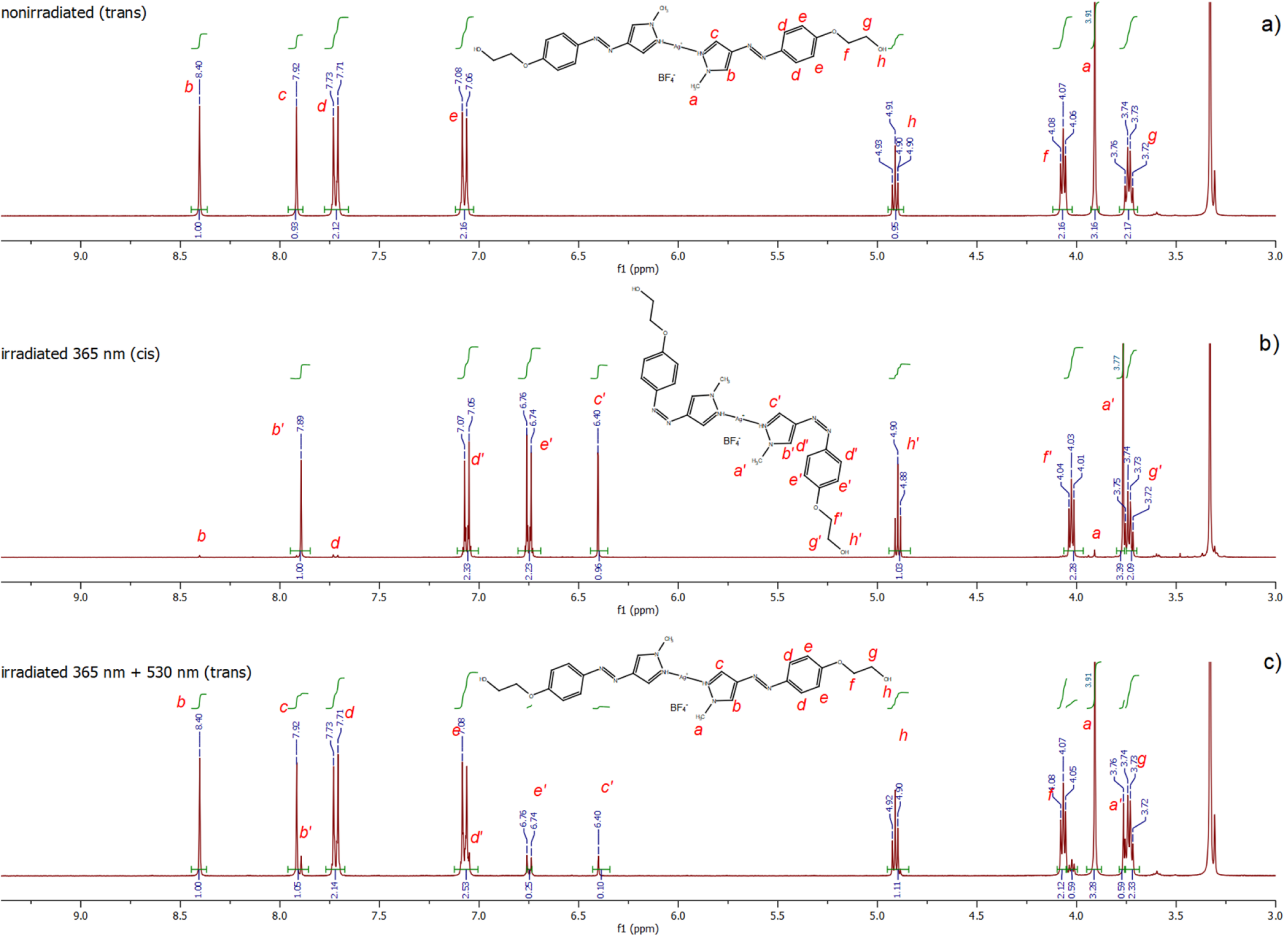
^1^H NMR (400 MHz, DMSO-d_6_) spectra of the complex (a) nonirradiated (*trans*), (b) irradiated with 365 nm light for 50 min (*cis*), and then (c) irradiated with 530 nm light for 150 min (mainly *trans*).

*Trans–cis* isomerization of the complex due to irradiation with 365 nm light resulted in the significant upfield shift of the pyrazole proton singlets (from 8.40 and 7.92 ppm to 7.89 and 6.40 ppm, respectively), the phenylene proton doublets (from 7.73, 7.71, 7.08, and 7.06 to 7.07, 7.05, 6.76, and 6.74 ppm, respectively), and a much smaller shift of the methyl singlet (from 3.91 to 3.77 ppm). The position of the ethylene protons was essentially unaffected by photoisomerization.

The signals of aromatic protons of Ag­(*trans*-PS1)_2_BF_4_ (Figure 5a) are practically absent in the spectrum of Ag­(*cis*-PS1)_2_BF_4_ (Figure 5b), which indicated that the *trans–cis* photoisomerization of the *trans-*PS1 ligands in the complex occurred with ≈100% yield. After irradiation of Ag­(*cis*-PS1)_2_BF_4_ with 530 nm light, the chemical shifts were restored to their original values, characteristic of Ag­(*trans*-PS1)_2_BF_4_, indicating the occurrence of the *cis–trans* photoisomerization (Figure 5c). The presence of small signals characteristic of Ag­(*cis*-PS1)_2_BF_4_ revealed, however, that the *cis–trans* photoisomerization was not quantitative but still high (about 91%), as was also observed in the case of the previously studied platinum complex with the same ligand.^34^

It was also revealed that Ag­(*trans*-PS1)_2_BF_4_ undergoes partial *trans–cis* photoisomerization under sunlight (Figure S8ab). In the PSS, which was established after 14 days of exposure of the Ag­(*trans*-PS1)_2_BF_4_ sample to summer daylight at room temperature, the content of this photoisomer was found to be about 62%. The spectrum of Ag­(*trans*-PS1)_2_BF_4_ kept in the dark did not change for at least 13 days (Figure S8c).

Typically, the *cis* isomers of the azo compounds are metastable and spontaneously isomerize in the dark to the *trans* isomers. The kinetic parameters of the thermal (dark) *cis–trans* isomerization of Ag­(*cis*-PS1)_2_BF_4_ were determined by using its UV–vis spectra measured at various temperatures in 1% v/v DMSO (Figure 6).

**Figure 6:**
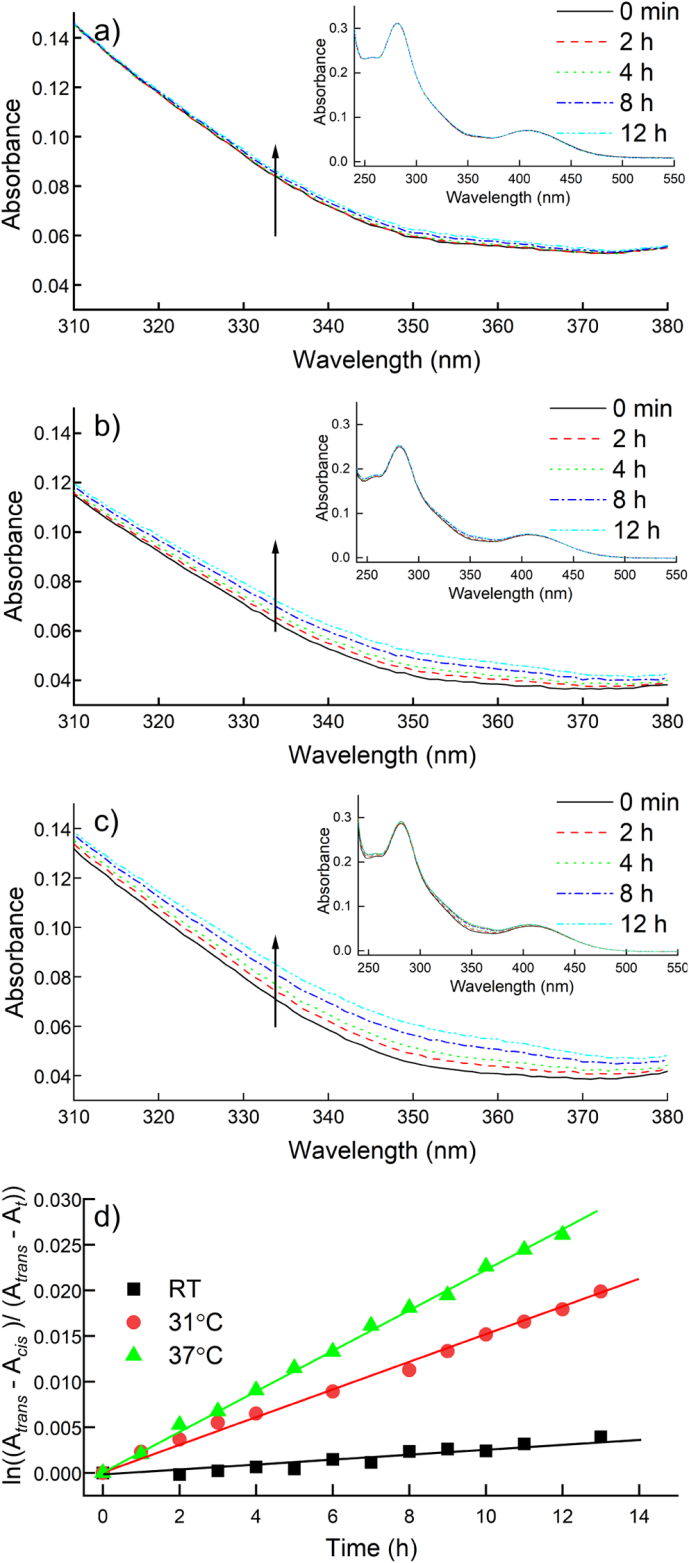
UV–vis spectra of Ag­(*cis*-PS1)_2_BF in 1% v/v DMSO measured after various times from sample preparation at (a) RT, (b) 31 °C, and (c) 37 °C (insets show full spectra). (d) First-order kinetic plots of Ag­(*cis*-PS1)_2_BF_4_→Ag­(*trans*-PS1)_2_BF_4_ thermal isomerization obtained from spectra are shown in panels a–c.

The obtained plots were linear, confirming the assumed first-order kinetics of the reaction (Figure 6d). Based on their slopes, the corresponding *cis–trans* isomerization rate constants, *k*, and the half-lives of the *cis* isomer at each temperature were calculated (Table 1). Using the Arrhenius equation, the activation energy value of 98.7 kJ/mol was determined (Figure S9). The half-life of the *cis* form of the complex in the aqueous medium at physiological temperature was equal to 311 h, or about 13 days. This was notably longer than the half-life reported for the free *cis*-PS1 (218 h or 9 days),^35^ so coordination of *cis-*PS1 with silver ion in Ag­(*cis*-PS1_2_)_2_BF_4_ enhanced its thermal stability. Due to the long half-life of Ag­(*cis*-PS1_2_)_2_BF_4_ the complex introduced to the cells may exist there in two different forms for a time long enough to reveal differences in their physiological activities, thus enabling potential photopharmacological applications of the complex.

**Table 1:** First-Order Thermal Isomerization Rate Constants for Ag­(*cis*-PS1)_2_BF_4_ in 1% v/v DMSO and Corresponding Half-Lives Obtained from Figure 6d

	**Temperature**		
	RT	31 °C	37 °C
***k*** **·** **10** ^**3**^ **[1/h]**	0.26 ± 0.02	1.51 ± 0.02	2.23 ± 0.02
***τ***_**1/2**_ **[h/d]**	2629/109	459/19	311/13

To investigate the photochemical stability of the complex against side photoreactions, such as photooxidation, during multiple back-and-forth *trans–cis* photoisomerizations, it was subjected to the repeated *trans–cis–trans* photoisomerization cycles, and the changes in the relative absorbance at the maximum of Ag­(*trans*-PS1)_2_BF_4_ absorption band (357 nm) were monitored. After 6 cycles, the absorbance at the maximum of the absorption band decreased by only ∼8% (Figure S10), indicating relatively high photochemical stability of the complex.

### Toxicity of Ag­(PS1)_2_BF_4_ in Cancer Cells

To find out if the photoisomerization of the complex brings about a meaningful change in its cytotoxicity in cancer cells, both its photoisomers were tested *in vitro* in selected tumor cell lines, i.e., human prostate cancer (PC3), human nonsmall cell lung cancer (A549), and murine mammary gland cancer cells (4T1). The complex toxicity was also assessed in normal murine mammary gland cells (NMuMG).

In PC3 cells, both photoisomers were nontoxic up to 8.3 μM (Figure 7a). At concentrations >33 μΜ, the *cis* isomer showed some toxicity, but it was not statistically different from that of the *trans* photoisomer. Also, in A549 cells, both photoisomers were nontoxic (Figure 7b); only at higher concentrations (>33 μM) did the *cis* photoisomer show slightly higher toxicity than the *trans* one. However, in murine mammary gland cells 4T1 (cancer, Figure 7c) and NMuMG (normal, Figure 7d), both photoisomers showed dose-dependent toxicity. In 4T1 cells at concentrations of 9.0–72 μM, there was also a significant difference between the toxicities of both photoisomers, with the *trans* photoisomer being much more toxic (Table 2). This difference was maximal at 72 μM, with the viability of the cells at this complex concentration being 25 times higher for the *cis* photoisomer than for the *trans* one. In NMuMG cells, the complex exhibited toxicity at concentrations >18.0 μM, with the *trans* photoisomer being notably more toxic (Table 2). The difference between *trans* and *cis* photoisomer toxicities was, however, not as pronounced as in the corresponding 4T1 cancer cells—the *cis*/*trans* viability ratio reached 6.3. Moreover, in the case of normal NMuMG cells, the range between nontoxic and highly toxic concentrations was rather narrow, i.e., 18–36 and 18–72 μM for the *trans* and *cis* photoisomers, respectively, while in the corresponding 4T1 cancer cells, the toxicity grew in a much wider concentration range (9–289 and 9–72 μM for the *cis* and *trans* photoisomers, respectively). Notably, there is a concentration window around 18 μM in which the *cis* photoisomer is not toxic in both normal and cancer mammary gland cells, while the *trans* photoisomer is toxic in cancer cells and nontoxic in normal cells. Advantageously, considering the LC50 values expressed in milligrams per milliliter, the more toxic *trans* photoisomer is about twice as toxic as cisplatin, a reference metal complex drug, while the *cis* photoisomer is about three times less toxic in the cancer 4T1 cells than cisplatin (Table 2). On the other hand, in the normal NMuMG cells, the toxicity of *trans* and *cis* photoisomers is about 1.5 and 2.7 times less than that of cisplatin, respectively.

**Figure 7:**
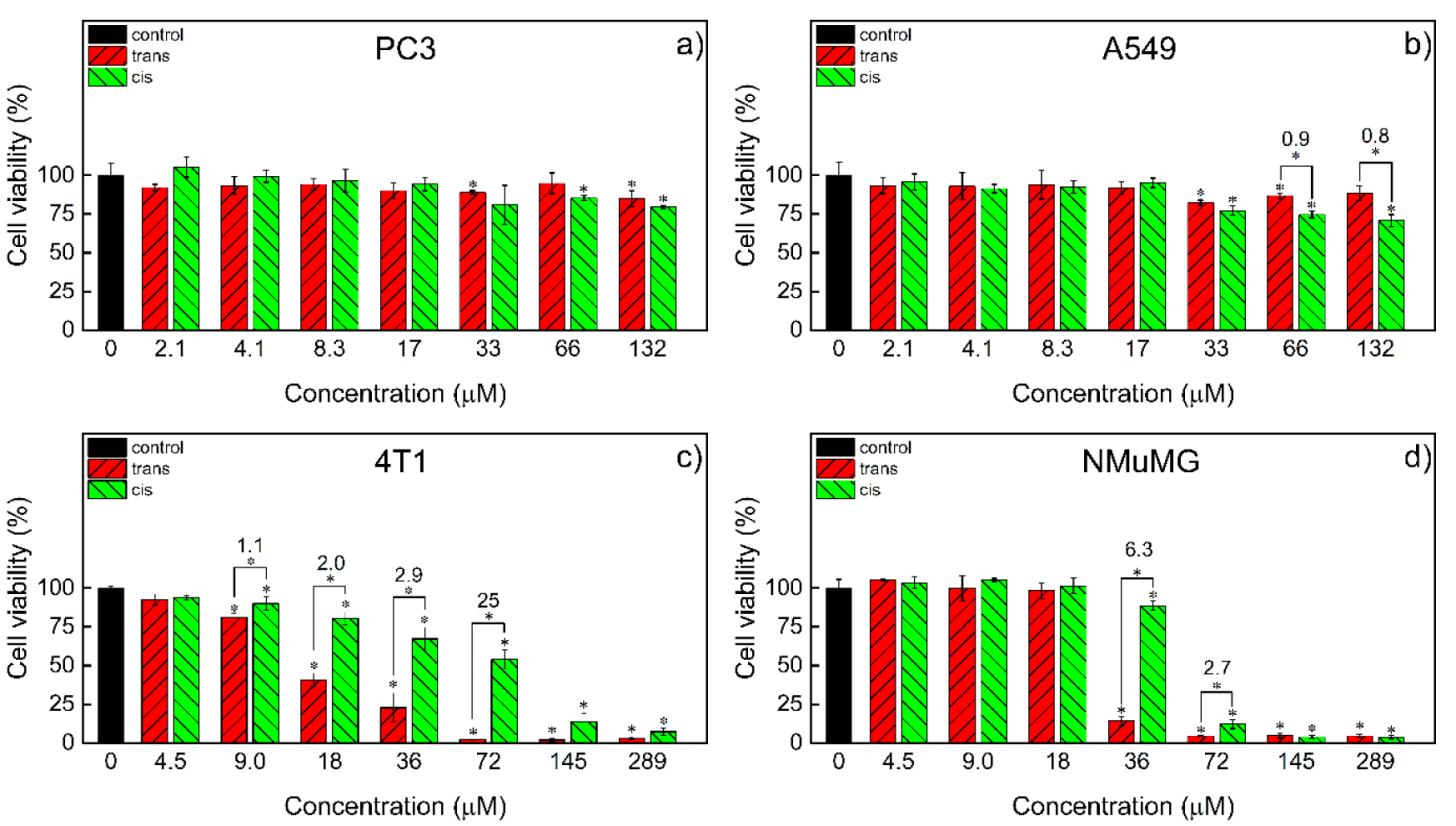
Cytotoxicity of trans and cis photoisomers of Ag­(PS1)_2_BF_4_ in PC3 (a), A549 (b), 4T1 (c), and NMuMG (d) cells after 24 h of cell culture determined with the MTT test. The numbers are the ratios of the viabilities of the cis and trans photoisomers at a given concentration. Statistically significant differences are indicated with asterisks above the bars (vs control) and above the brackets (trans vs cis), *n* = 3, Mann–Whitney test, *p* = 0.05.

**Table 2:** LC50 Values of Both Photoisomers in Cancer (4T1) and Normal (NMuMG) Murine Mammary Gland Cells Found by Fitting Hill’s Equation (Figure S11) to the Cell Viability Data Shown in Figures 7c and 8d^a^

	**LC50 (** $\mathrm{\mu M}/\frac{\mathrm{\mu g}}{\mathrm{mL}}$ **)**	
Compound	4T1	NMuMG
Ag(*trans*-PS1)_2_BF_4_	16/11	26/18
Ag(*cis*-PS1)_2_BF_4_	100/69	47/33
Cisplatin	71/21 ^34^	40/12 ^34^

a Toxicity of cisplatin was used for comparison.

Different toxicity of the complex in the cell lines studied (nontoxic in PC3 and A549, toxic in 4T1 and NMuMG cells) may reflect differences in cellular uptake efficiency, membrane composition, and presence/absence of specific receptors and transporters (Ag^+^ ions are transported by the high-affinity copper transporter 1, CTR1). Different levels of cellular thiols (GSH/GSSG, cysteine in proteins) responsible for the cellular redox equilibrium and the resistance to the oxidative stress may also contribute to these differences.

### Photoswitching of the Cytotoxicity of Ag­(PS1)_2_BF_4_ upon Uptake by the NMuMG Cells

The difference in the toxicity of both photoisomers of the complex may stem from both their intrinsic toxicity and the difference in their uptake by the cells. To estimate their intrinsic toxicity, the NMuMG cells were incubated for 0.5 and 1.0 h in the medium containing Ag­(*cis-*PS1)_2_BF_4_. Then, the culture medium was replaced with a clean one; half of the cells were irradiated with 530 nm light for 5 min in the incubator to transform the complex into the more toxic *trans* isomer, and the other half were cultured in the dark. These culture conditions ensured that the complex underwent photoisomerization only after internalization. Irradiation resulted in a decrease of the cell viability by 22 and 55% for uptake times of 0.5 and 1 h, respectively (Figure 8), so the relative change in the cell viability is much larger for the longer incubation time. These results confirm that it is possible to activate the complex already taken up by the cells with 530 nm. Moreover, the difference in the toxicity of both photoisomers is smaller than for NMuMG cultured in the medium containing the complex for 24 h, probably because the uptake of both photoisomers is different, as was also determined for the analogical complex of platinum.^34^

**Figure 8:**
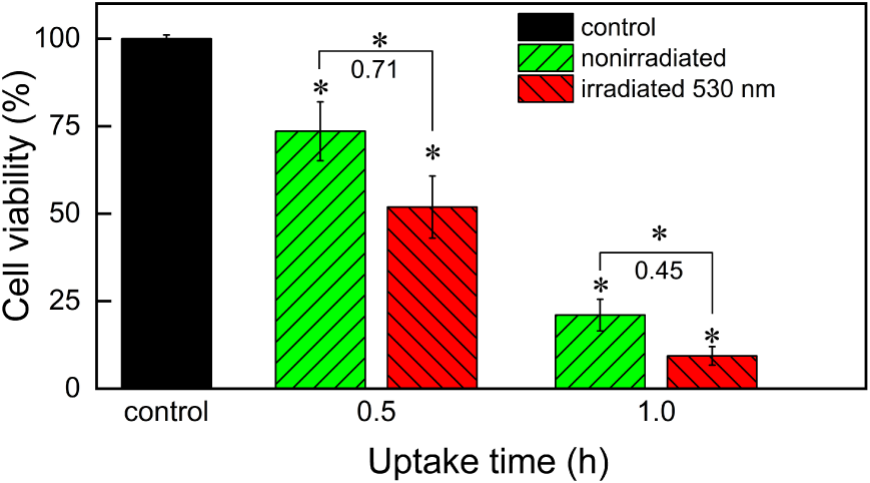
Cytotoxicity of Ag­(*cis-*PS1)_2_BF_4_ nonirradiated and irradiated with 530 nm light upon internalization by NMuMG cells after 24 h of culture determined by the MTT test. The cells were irradiated directly after completion of the complex uptake for 5 min with 530 nm light to convert the *cis* photoisomer into the more toxic *trans* one. The numbers denote the irradiated/nonirradiated cell viability ratios for the respective uptake time. Statistically significant differences are indicated with asterisks above the bars (vs control) and above the brackets (nonirradiated vs irradiated photoisomers), *n* = 3, Mann–Whitney test, *p* = 0.05.

### Assessment of Antibacterial and Antifungal Activity of Ag­(PS1)_2_BF_4_

The antibacterial properties of the complex were tested using two model bacterial strains, the Gram-positive *Staphylococcus aureus* and the Gram-negative *Escherichia coli*, while its antifungal activity was assessed using *Candida albicans* yeast and spore-forming filamentous *Aspergillus fumigatus*. Ag­(PS1)_2_BF_4_ exhibited both antibacterial and antifungal *in vitro* activity against all of the tested microbial strains. Minimum inhibitory concentration (MIC) values are given in Table 3. Minimum bactericidal concentration (MBC) and minimum fungicidal concentration (MFC) values, however, could not be determined, as the complex, even at the highest concentration tested, did not completely eradicate microbial growth on solid media, indicating a predominantly inhibitory rather than cidal mode of action.

**Table 3:** Visual Minimum Inhibitory Concentration (MIC) Values of Ag­(PS1)_2_BF_4_ against Selected Microorganisms

Microorganism	MIC μM (mg/L)
*Staphylococcus aureus*	72.76 (50) - both *trans* and *cis*
*Escherichia coli*	72.76 (50) - both *trans* and *cis*
*Candida albicans*	18.19 (12.5) - both *trans* and *cis* starting from 1.14 (0.78) gradual growth inhibition was observed
*Aspergillus fumigatus*	4.54 (3.12) - both *trans* and *cis*

The MIC values obtained for both fungal strains were significantly lower than those observed for both bacterial strains, indicating that the complex displays stronger antifungal than antibacterial activity. Importantly, no significant differences were observed in antimicrobial activity between the *trans* and *cis* photoisomers of the complex.

Since Ag­(PS1)_2_BF_4_ demonstrated growth-inhibitory effects against fungal strains, its potential for topical application in the treatment of fungal skin infections was explored. To evaluate its safety in this context, the cytotoxicity of the isomers toward human skin keratinocytes (HaCaT) was assessed using the MTT assay. Ag­(*cis*-PS1)_2_BF_4_ exhibited no toxicity toward HaCaT cells across the entire concentration range studied (Figure 9a), while Ag­(*trans*-PS1)_2_BF_4_ reduced HaCaT cell viability at concentrations above 9.1 μM after 24 h of exposure. At the highest complex concentration studied (73 μM), the ratio of HaCaT cell viabilities for *cis* and *trans* photoisomers was 17 (Figure 9), indicating a significant difference in their toxicities in HaCaT cells. The LC50 value of Ag­(*trans*-PS1)_2_BF_4_ was about 31 μM, as found from fitting the experimental data to Hill’s equation (Figure 9b), which was 2.5 times higher than the MIC values for Ag­(*trans*-PS1)_2_BF_4_ against *Candida albicans* and 10 times higher than the MIC against *Aspergillus fumigatus* for that isomer (Table 3). Thus, one can identify concentration ranges at which *cis* (>4.54 μM) and *trans* isomers (4.54–31 μM) of Ag­(PS1)_2_BF_4_ exhibit inhibitory effects against *Aspergillus fumigatus* and at the same time remain nontoxic to keratinocytes following 24-h incubation. Interestingly, it was previously demonstrated that ionic silver at the subcytotoxic concentrations actually promotes the proliferation of human keratinocytes, which may be important in planning the therapeutic process.^39^

**Figure 9:**
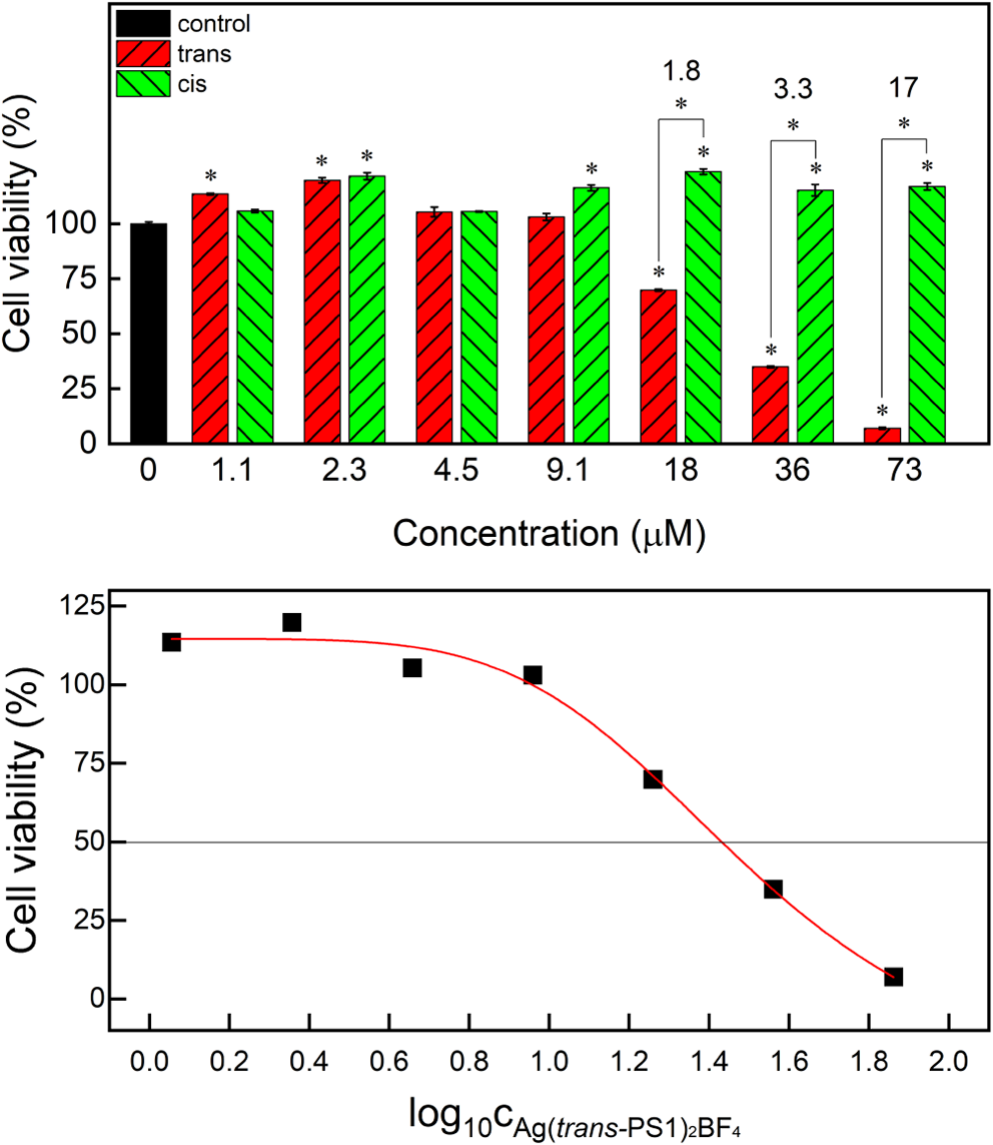
(a) Cytotoxicity of both photoisomers of Ag­(PS1)_2_BF_4_ in HaCaT cells after 24 h of culture. The numbers denote the ratio of the viabilities of the *cis* and *trans* photoisomers for a given concentration. Statistically significant differences are indicated with asterisks above the bars (vs control) and above the brackets (*trans* vs *cis*), *n* = 3, Mann–Whitney test, *p* = 0.05. (b) The experimental data fitted to Hill’s equation used to find the LC50 of Ag­(*trans*-PS1)_2_BF_4_.

## Conclusions

The novel silver­(I) complex containing two photoswitchable ligands, Ag­(PS1)_2_BF_4_, was synthesized. The complex crystallizes in monoclinic space group *P*2_1_/*c*, with one Ag^+^ ion, two *trans-*PS1 ligands, and one BF_4_^–^ anion in the asymmetric unit. The two photoswitchable ligands undergo reversible photoisomerization when irradiated with 365 (*trans–cis*) and 530 nm light (*cis–trans*). The lifetime of the thermally unstable *cis* photoisomer is about 13 days, enabling photopharmaceutical applications of the complex. *In vitro* cytotoxicity tests have demonstrated that the complex showed dose-dependent photoswitchable toxicity (significantly higher for the *trans* photoisomer) in murine mammary gland cells. It was, however, not toxic in human prostate cancer PC3 and nonsmall cell lung cancer A549 cells. The complex was also shown to have bacteriostatic and fungistatic properties; however, no significant difference between the activity of *trans* and *cis* photoisomers was found. In human keratinocytes, the *cis* photoisomer was nontoxic, while the toxicity of the *trans* photoisomer was much lower than its MIC in *Aspergillus fumigatus* opening the perspective for Ag­(PS1)_2_BF_4_ application in the treatment of fungal infections.

## Experimental Section

### Reagents

AgBF_4_ (Merck), anhydrous THF (Thermo Fisher Scientific, 99.5%), diethyl ether (Chempur, analytical grade), formic acid (Sigma-Aldrich), DMSO (Sigma-Aldrich), pH 7.4 PBS tablets (Sigma-Aldrich), DMSO-d_6_ (Deutero GmbH, 99.8%), MTT reagent (3-[4,5-dimethylthiazol-2-yl]-2,5-diphenyltetrazolium bromide, AmBeed), Dulbecco’s modified Eagle’s medium high glucose (DMEM, Sigma-Aldrich), fetal bovine serum (FBS, Sigma-Aldrich), trypsin-EDTA solution (Sigma-Aldrich), penicillin (Sigma-Aldrich), streptomycin (Sigma-Aldrich), and DMSO cell culture grade (Apollo Scientific) were used as received.

### Apparatus

NMR spectra of the complex were recorded in DMSO-d_6_ using JEOL spectrometers operating at 400 and 600 MHz. ATR IR spectra were obtained by using a Nicolet iS10 spectrophotometer (Thermo Scientific). UV–Vis absorption spectra were measured with a Varian Cary 50 UV–Vis spectrophotometer (Agilent Technologies). Irradiation experiments were performed using 365 and 530 nm LED plates (BioResearch Center, Japan). Cytotoxicity assays were measured using a Synergy HTX microplate reader (BioTek, Winooski, VT, USA).

### Synthesis of Ag­(trans-PS1)_2_BF_4_

Synthesis of (E)-2-(4-((1-methyl-1H-pyrazol-4-yl)­diazenyl)­phenoxy)­ethan-1-ol (*trans-*PS1) was performed according to the procedure described in our previous paper.^35^ The synthesis of Ag­(*trans-*PS1)_2_BF_4_ was performed based on a modified literature procedure.^36^ Briefly, to 500 mg (2 eq., 2.03 mmol) of *trans-*PS1 dissolved in 48 mL dry THF, 197.62 mg (1 eq., 1.00 mmol) of AgBF_4_ dissolved in 57 mL dry THF was added. The mixture was protected from light and purged with argon for 15 min. It was stirred without access to oxygen for 72 h. Then, the precipitated product was centrifuged (10 min, 10 000 rpm), and the supernatant was removed. Ten mL of THF was added, the suspension was mixed, and the supernatant was decanted. The washing procedure was repeated four times. Next, the product was washed twice with 10 mL of diethyl ether and dried at 20 °C for 24 h in a vacuum oven. Yield: 475 mg (69%).

### Single-Crystal X-ray Diffraction (SCXRD) Measurements and Structure Refinement

SCXRD experiments were performed at T = 293(2) K (Table S2) using an Oxford XtaLAB Synergy-S X-ray diffractometer (PS1) and at T = 291(2) K using an Oxford Diffraction Gemini R ULTRA Ruby CCD diffractometer (Ag­(PS_1_)_2_BF_4_) (λ_Cu_ = 1.54184 Å).^40^ The weakly diffracting crystal of Ag­(PS_1_)_2_BF_4_ shows no detectable diffraction below a θ angle of 62.5° (with a ratio of observed to unique reflections of 30%), which results in a high R1 value. The structures were solved and refined using the SHELX package programs.^41,42^ H atoms from hydroxyl groups were located on a difference Fourier map and refined with U_iso_(H) = 1.5U_eq_(O), whereas H atoms bound to C atoms were placed geometrically and refined using a riding model with C–H = 0.93–0.97 Å and U_iso_(H) = 1.2U_eq_(C) (C–H = 0.96 Å and U_iso_(H) = 1.5U_eq_(C) for the methyl groups). The BF_4_^–^ anion is disordered over two orientations, with refined site-occupancy factors of 0.78(2) and 0.22(2) for the respective components, and was refined as rigid, ideally tetrahedral units (the total number of restraints applied was 23). The BF_4_^–^ anion exhibits disorder over two orientations, with refined site occupancy factors of 0.78(2) and 0.22(2) for the respective components. In Figure S5 presenting the crystal structure, the disordered part of the BF_4_^–^ anion is omitted for clarity. All interactions in the complex were found using the PLATON program.^43^ The ORTEPII^44^ and Mercury^45^ programs were used to prepare molecular graphics.

### Assessment of Antibacterial and Antifungal Activity

The *in vitro* antimicrobial activity of Ag­(PS_1_)_2_BF_4_ against selected bacteria and fungi was performed using a microdilution method in liquid culture media based on the European Committee on Antimicrobial Susceptibility Testing methodology for fungi^46,47^ with some modifications in the case of bacteria.

The bacterial strains were purchased from the American Type Culture Collection (ATCC): *Staphylococcus aureus* ATCC 29213 and *Escherichia coli* ATCC 15922, and the fungal strains from the German Collection of Microorganisms and Cell Cultures (DSMZ): *Candida albicans* DSM 11225 and *Aspergillus fumigatus* DSM 819. The strains were stored frozen at −72 °C and revived immediately prior to testing. Bacterial inocula were prepared from 24 h cultures on Tryptic Soy Agar (Becton-Dickinson) by suspending a few colonies in sterile distilled water to obtain 0.5 McFarland cell density and later diluted 300-fold in sterile water. Fungi were cultured on Sabouraud glucose agar with chloramphenicol (Difco Laboratories Inc., Franklin Lakes, NJ, USA) to obtain optimal growth and sporulation. *C. albicans* inoculum was prepared by suspending a few representative colonies in sterile distilled water. *A. fumigatus* colonies were covered with approximately 5 mL of sterile water supplemented with Tween 20 (Sigma-Aldrich, St. Louis, MO, USA), and the conidia were rubbed with a sterile cotton swab. The suspension was collected in a sterile tube attached to a sterile filter with a pore diameter of 10 μm to remove hyphal fragments. Fungal suspensions were homogenized with a gyratory vortex mixer, and the cell density was adjusted to 0.5 McFarland. Such inocula were diluted 1:20 with sterile water.

The tested complex was dissolved in DMSO to obtain an initial concentration of 40000 mg/L. This solution was used to prepare a series of 9 subsequent 2-fold dilutions using DMSO as the diluent. Finally, 10 serial concentrations of Ag­(PS1)_2_BF_4_ were diluted 1:100 in double-strength culture media—Miller Hinton Broth II (Merck, Germany) for bacteria and RPMI-1640 medium with l-glutamine, without sodium bicarbonate (Sigma-Aldrich, USA), supplemented with 2% glucose (Chempur) and buffered to pH 7 with 4-morpholinepropanesulfonic acid (MOPS; 0.165 mol/L) (Glentham Life, Corsham, UK) for fungi. This resulted in concentrations of the complex ranging from 0.78 to 400 mg/L.

Incubation of microorganisms in the presence of the complex was carried out in flat-bottom polystyrene 96-well microdilution plates (Nest, China). Microdilution plates were filled with 100 μL of the corresponding concentration of the examined substance in double-strength media and inoculated with 100 μL of microbial suspensions. The final microbial inocula concentrations on the microtiter plates were approximately 2.5 × 10^5^, (0.5–2.5) × 10^5^, and (1–2.5) × 10^5^ CFU/mL for bacteria, *C. albicans*, and *A. fumigatus*, respectively. Final concentrations of the complex ranged from 0.39 to 200 mg/L. Immediately after the inoculation, some of the plates were irradiated for 20 s with 365 nm light. The tests were performed in triplicate.

The plates were incubated without agitation at 35 ± 2 °C for bacteria and *C. albicans*, and at 27 ± 2 °C for *A. fumigatus* in ambient air for 24 h. The readings were taken visually and with a microdilution plate reader (Tecan, Sunrise) measuring the absorbance at a 530 nm wavelength. Antifungal activity of Ag­(PS1)_2_BF_4_ was estimated by determining the minimal inhibitory concentration (MIC) values, which were defined as no visible growth of fungi as observed by the naked eye.

After visual and automatic readings, the contents of the wells with no visible growth to the naked eye were transferred onto solid media—Tryptic Soy Agar (Becton-Dickinson) for bacteria and Sabouraud glucose agar with chloramphenicol for fungi—to determine the minimal bactericidal concentrations (MBC) and minimal fungicidal concentrations (MFC). The inoculated media were incubated at 35 ± 2 °C for bacteria and *C. albicans*, and at 27 ± 2 °C for *A. fumigatus* in ambient air for 24 h. After incubation, the results were assessed visually for the presence of microbial colonies. MBC and MFC values were defined as the lowest concentrations of the tested compound for which no microbial growth was observed in the medium.

### Cytotoxicity Tests

Cytotoxicity studies of the complex and ligand were performed on both normal and cancerous cell lines: normal mouse mammary gland epithelial cells (NMuMG, ATCC CRL-1636), murine mammary carcinoma cells (4T1, ATCC CRL-2539), human nonsmall cell lung cancer (A549, ATCC CCL-185), human prostate cancer (PC3, ATCC CRL-1435), and human keratinocyte (HaCaT, ThermoFisher). Cells were cultured in Petri dishes using high-glucose DMEM. For NMuMG cells, 10 μg/mL insulin was additionally supplemented. All media were supplemented with 10% (v/v) fetal bovine serum (FBS) and incubated at 37 °C in a humidified atmosphere containing 5% (v/v) CO_2_. Next, cells were seeded into 96-well plates and incubated for 24 h. They were then treated with various concentrations of both photoisomers of the complex, dissolved in a 1% (v/v) DMSO solution in culture medium (with the appropriate serum content). Medium without the complex was used as a control. Stock solutions of the complexes were prepared in DMSO, immediately diluted with medium, and promptly added to the cell cultures. Incubation period was 24 h. Following treatment, the medium was removed, and 100 μL of MTT solution (3-[4,5-dimethylthiazol-2-yl]-2,5-diphenyltetrazolium bromide) in medium was added to each well and incubated in the dark for 4 h at 37 °C. The MTT solution was then gently aspirated, and 100 μL of a 1:1 (v/v) DMSO/isopropanol mixture was added to dissolve the resulting purple formazan crystals. Absorbance was measured at 570 nm by using a plate reader. All measurements were performed in triplicate. Statistical significance was determined at *p* = 0.05.

## Supplementary Material



## Notes

Elemental analysis of the complex, crystallographic data on PS1 and Ag­(*trans*-PS1)_2_BF_4_, geometry of weak interactions in PS1, hydrogen bonds in Ag­(*trans*-PS1)_2_BF_4_, and π–π stacking interactions geometry for Ag­(*trans*-PS1)_2_BF_4_, ^1^H NMR, UV–vis, and ATR IR spectra of Ag­(*trans*-PS1)_2_BF_4_ and *trans*-PS1, UV–vis spectra of Ag­(*trans*-PS1)_2_BF_4_ irradiated with 400 nm, photostationary distribution at various irradiation wavelengths, kinetics of *trans–cis* and *cis–trans* photoisomerizations, ^1^H NMR spectra of Ag­(*trans*-PS1)_2_BF_4_ in DMSO-d_6_ nonirradiated, irradiated with daylight for 14 days, and nonirradiated 13 days after preparation, activation energy of dark *cis–trans* isomerization, stability of the complex studied with UV–vis spectroscopy, Hill plots for the cytotoxicity of both complex isomers in 4T1 and NMuMG cell lines (PDF)

## #

Selvita S.A, Bobrzyńskiego 14, 30–348 Kraków, Poland
