# Supplementary material for: Photoswitchable Silver(I) Complex with Anticancer and Antimicrobial Potential
Source: ACS Omega. 2026 Mar 29;11(13):21162–72. doi: 10.1021/acsomega.6c00430 (PMC13063025; doi:10.1021/acsomega.6c00430)
Supplement: Supplementary file 1 [file ao6c00430_si_001.pdf]

Photoswitchable Silver(I) Complex with Anticancer and Antimicrobial Potential

Supporting Information

Aleksandra Kręcigłowa[1,‡], Marta Stolarek-Sipior[1,2,‡], Patrycja Jagielska[1,2], Kamil Kamiński[1], Magdalena Skóra[3], Marlena Gryl, Artur Sikorski[4], Janusz Rak[4], Maria Nowakowska[1], and Krzysztof Szczubiałka[1,]*

[1] *Faculty of Chemistry, Jagiellonian University, Gronostajowa 2, 30-387 Cracow, Poland*
[2] *Doctoral School of Exact and Natural Sciences, Jagiellonian University, Łojasiewicza 11, 30-348 Cracow, Poland*
[3] *Department of Infection Control and Mycology, Jagiellonian University Medical College, Czysta 18, 31-121 Cracow, Poland*
[4] *Faculty of Chemistry, University of Gdansk, Wita Stwosza 63, 80-308 Gdansk, Poland*

‡ These authors contributed equally to this work.

Corresponding author email: k.szczubialka@uj.edu.pl

Table S1. Elemental analysis of the Ag(*trans*-PS1)$_2$BF$_4$

Element	Content in the complex (%)		Theoretical content in *trans*-PS1 (%)
	Theoretical	Experimental (results of 3 analyses and their average)	
**C**	41.95	41.22; 41.32; 41.33<br>41.29	58.53
**H**	4.11	3.93; 4.00; 3.90<br>3.94	5.73
**N**	16.31	15.52; 15.74; 15.41<br>15.56	22.75

Table S2. Crystal data and structure refinement for *trans*-PS1 and Ag(*trans*-PS1)$_2$BF$_4$.

**Compound**	***trans*-PS1**	**Ag(*trans*-PS1)$_2$BF$_4$**
Chemical formula	$C_{12}H_{14}N_3O_2$	$C_{24}H_{28}N_8O_4AgBF_4$
FW/g·mol^{-1}	232.26	687.22
Crystal system	orthorhombic	monoclinic
Space group	*P*bcn	$P2_1$/c
a/Å	53.3249(16)	18.698(5)
b/Å	7.0974(2)	7.1652(9)
c/Å	6.4747(2)	21.778(6)
α/°	90	90
β/°	90	104.76(3)
γ/°	90	90
V/Å^3	2450.47(13)	2821.4(12)
Z	8	4
T/K	293(2)	291(2)
λ/Å	1.54184	1.54184
ρ_{calc}/g·cm^{-3}	1.335	1.618
$F_{(000)}$	1040	1392
μ/mm^{-1}	0.780	6.381
θ range/°	3.32-79.58	4.20-62.50
Size of the crystal [mm]	0.40×0.15×0.02	0.31×0.17×0.10
Completeness of θ/%	97	99.5
Reflections collected	8472	15531
Reflections unique	2484	4480
Data/restraints/parameters	2484 / 1 / 167	4480 / 23 / 402
Goodness of fit on F^2	1.067	1.089
Final R_1 value (I>2σ(I))	0.0502	0.0957
Final wR_2 value (I>2σ(I))	0.1391	0.2062
Final R_1 value (all data)	0.0593	0.2134
Final wR_2 value (all data)	0.1456	0.2977
Largest diff. peak and hole/ e·Å^{-3}	-0.183/0.235	0.450 and -1.015
CCDC deposit number	2303673	2514396

Table S3. Geometry of weak interactions in PS1 [Å/°]

**D–H···A**	***d*(D–H)**	***d*(H···A)**	***d*(D···A)**	**∠D–H···A**
**C13-H13A···O1#1**	0.97	2.65	3.539(3)	152.7
**C11-H11···Cg1#2 (C6^C11)**	0.93	2.89	3.602	134.4
**C4-H4···Cg2#2 (N1^N2)**	0.93	2.90	3.690	143.8
**C7-H7···Cg3#3 (C6^C11)**	0.93	2.89	3.596	133.7
Symmetry transformations used to generate equivalent atoms: #1 -x+1,y,-z-1/2; #2 x, 2-y, ½+z; 3# x, 1-y, -1/2+z				

Table S4. Geometry of hydrogen bonds in the crystal structure of Ag(*trans*-PS1)$_2$BF$_4$ [Å/°]

D–H···A	*d*(D–H)	*d*(H···A)	*d*(D···A)	∠D–H···A
**O17–H17A···O37^{i}**	0.82	1.93	2.75(2)	176
**O37–H37A···F2^{ii}**	0.82	2.14	2.89(2)	151
**C16–H16A···F4^{iii}**	0.97	2.51	3.47(2)	172
**C18–H18B···F3^{iv}**	0.96	2.33	2.91(2)	119
**C25–H25A···F1^{iv}**	0.93	2.46	3.14(2)	130

Symmetry code: (i) -1+x,-1+y,z; (ii) 1+x,1/2-y,-1/2+z; (iii) x,1/2-y,-1/2+z; (iv) 1-x,1-y,-z.

Table S5. *π*–*π* stacking interactions geometry for Ag(*trans*-PS1)$_2$BF$_4$.

CgI[a]	CgJ[a]	CgI···CgJ[b] [Å]	Dihedral angle[c] [°]	Interplanar distance[d] [Å]
**Cg1**	Cg2^{v}	3.543(11)	4.1(1)	3.311(8)
**Cg1**	Cg3^{vi}	3.463(10)	0.7(1)	3.515(7)
**Cg2**	Cg4^{vii}	3.709(11)	6.8(1)	3.832(8)
Symmetry code: (v) x. -1+y, z; (vi) x, 1+y, z; (vii) 3-x.4-y,-z.				

(a) Cg represents the centre of gravity of the rings as follows: Cg1 ring N1/N2/C3/C4/C5, Cg2 ring N11/N12/C13/C14/C15, Cg3 ring C8/C9/C10/C11/C12/C13, Cg4 ring C28/C29/C30/C31/C32/C33, (b) Cg···Cg is the distance between ring centroids, (c) The dihedral angle is that between the mean planes of Cg(I) on ring J, (d) The interplanar distance is the perpendicular distance from CgI to ring J.

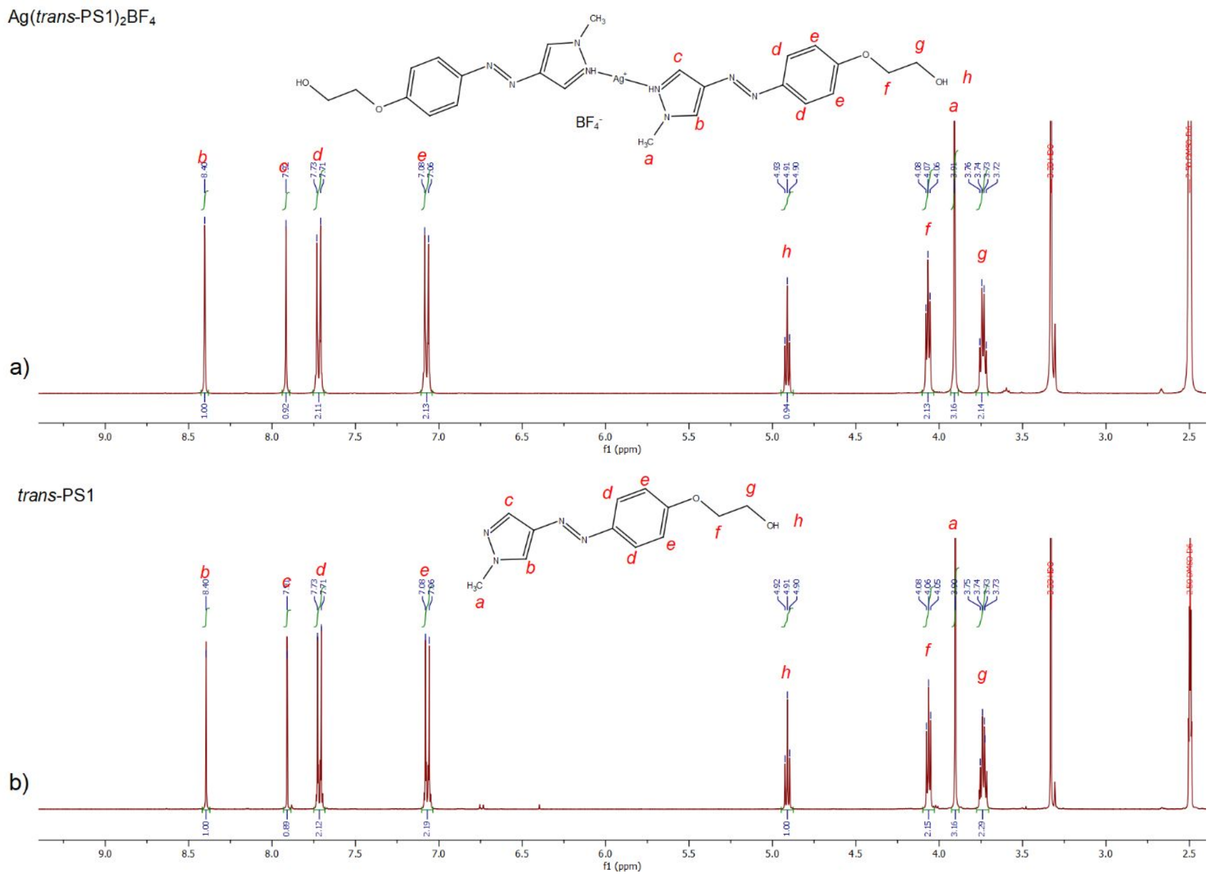

Figure S1. 1H NMR spectra of Ag(*trans*-PS1)$_2$BF$_4$ (a) and *trans*-PS1(b) in DMSO-d6.

Ag(*trans*-PS1)$_2$BF$_4$: ^{1}H NMR (400 MHz, DMSO-D_6) δ 8.40, 7.92, 7.73, 7.71, 7.08, 7.06, 4.91, 4.08, 4.07, 4.06, 3.91, 3.76, 3.74, 3.73, 3.33, 2.50.

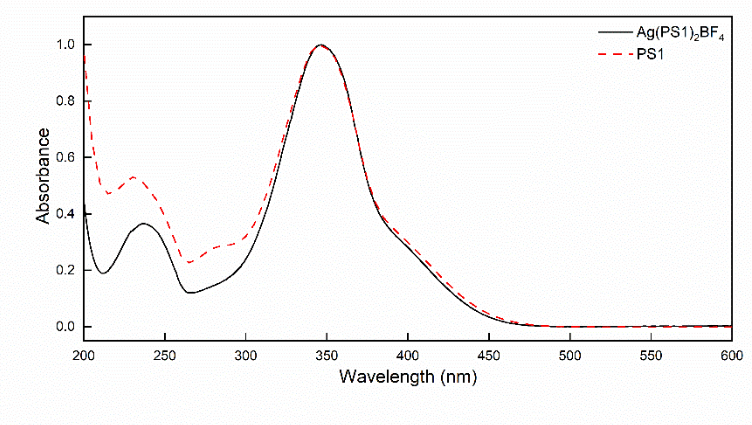

Figure S2. UV-Vis spectra of Ag(PS1)$_2$BF$_4$ (solid line) and PS1 (dashed line).

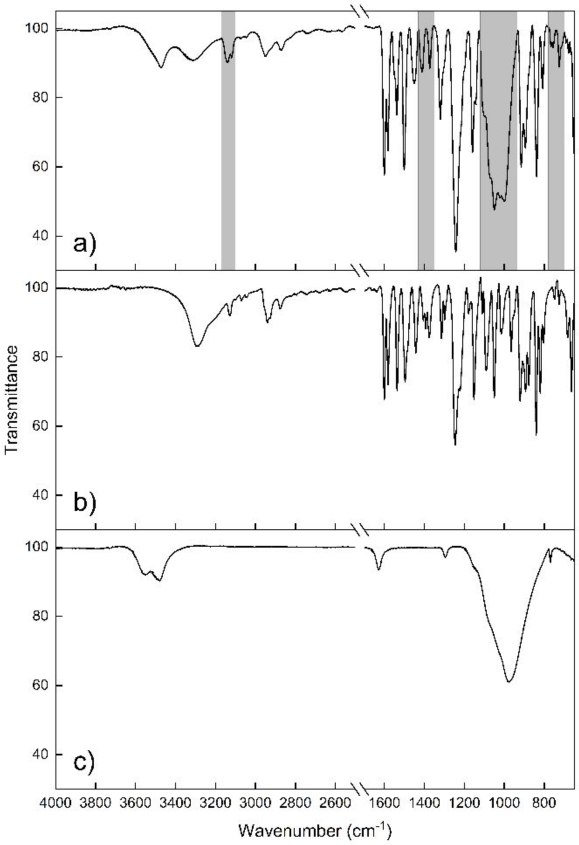

Figure S3. ATR IR spectra of Ag(*trans*-PS1)$_2$BF$_4$ (the bands most affected by the Ag(I) coordination are highlighted) (a), *trans*-PS1 (b), and AgBF$_4$ (c).

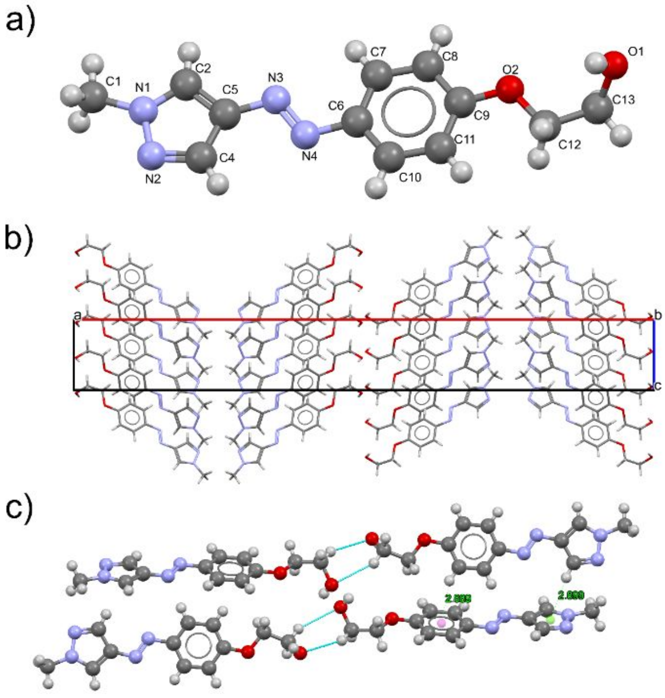

Figure S4. a) Asymmetric unit of PS1, b) structural components viewed along [010], and c) intermolecular interactions.

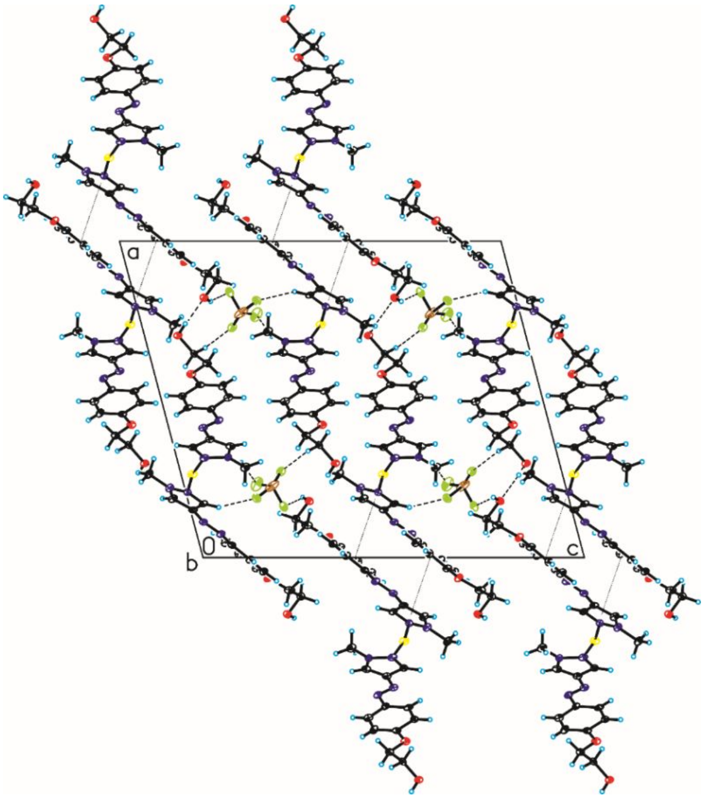

Figure S5. Crystal packing of Ag(*trans*-PS1)$_2$BF$_4$ viewed along the *b*-axis (hydrogen bonds are shown as dashed lines, whereas π–π interactions as dotted lines).

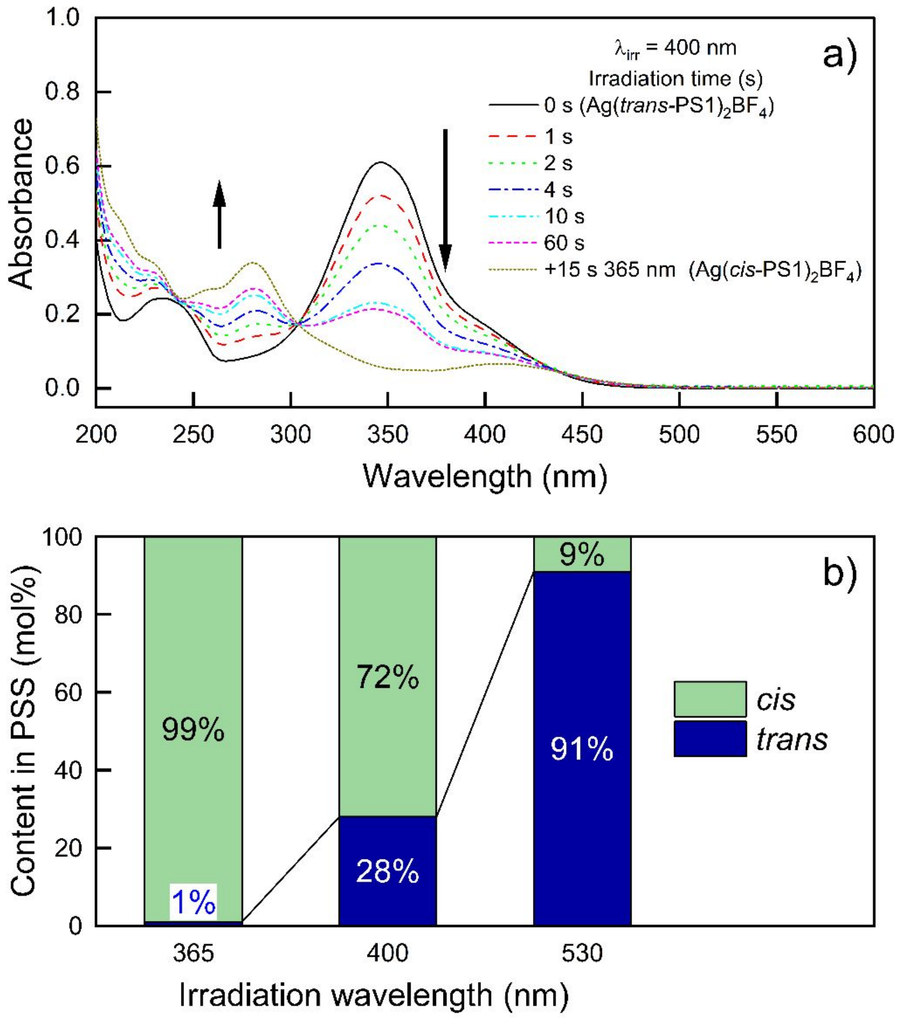

Figure S6. a) UV-Vis spectra of Ag(*trans*-PS1)$_2$BF$_4$ irradiated at RT in H_2O (0.0252 mM) with 400 nm light b) content of *trans* and *cis* photoisomers in the PSSs established after irradiation of the complex solution with light of various wavelengths. The power of the lamps was 51 and 26 mW for 400 and 365 nm lamp, respectively. Distance between the lamp and the cuvette: 10 cm.

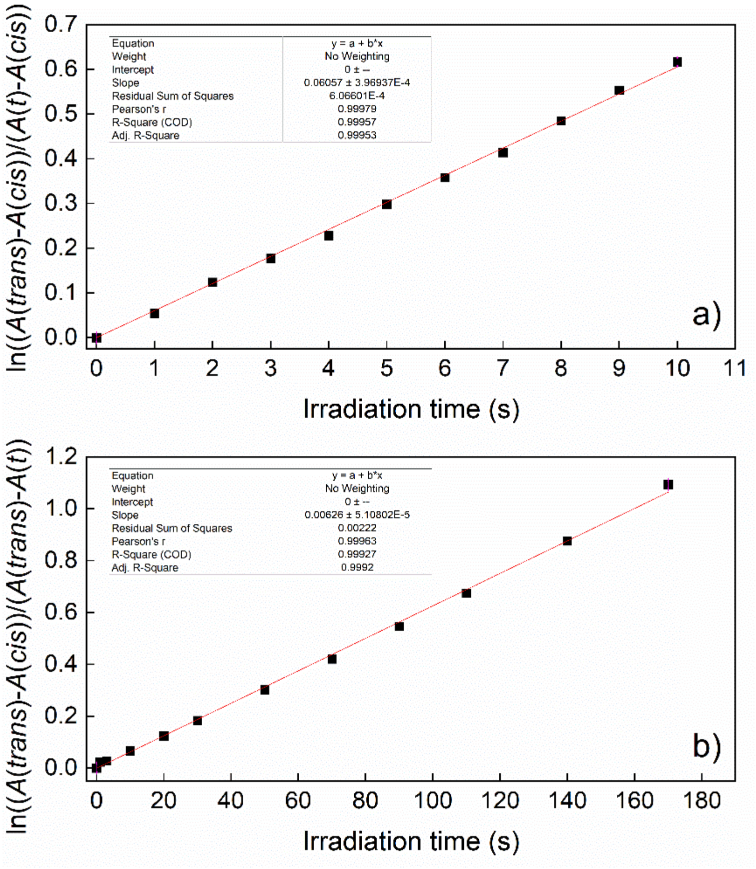

Figure S7. First-order kinetic plots for the *trans-cis* (a) and *cis-trans* (b) photoisomerizations of $Ag(PS1)_2BF_4$ at RT. The power of the 365 and 530 nm lamp was 26 and 22 mW, respectively. The distance between the lamp and the cuvette: 10 cm.

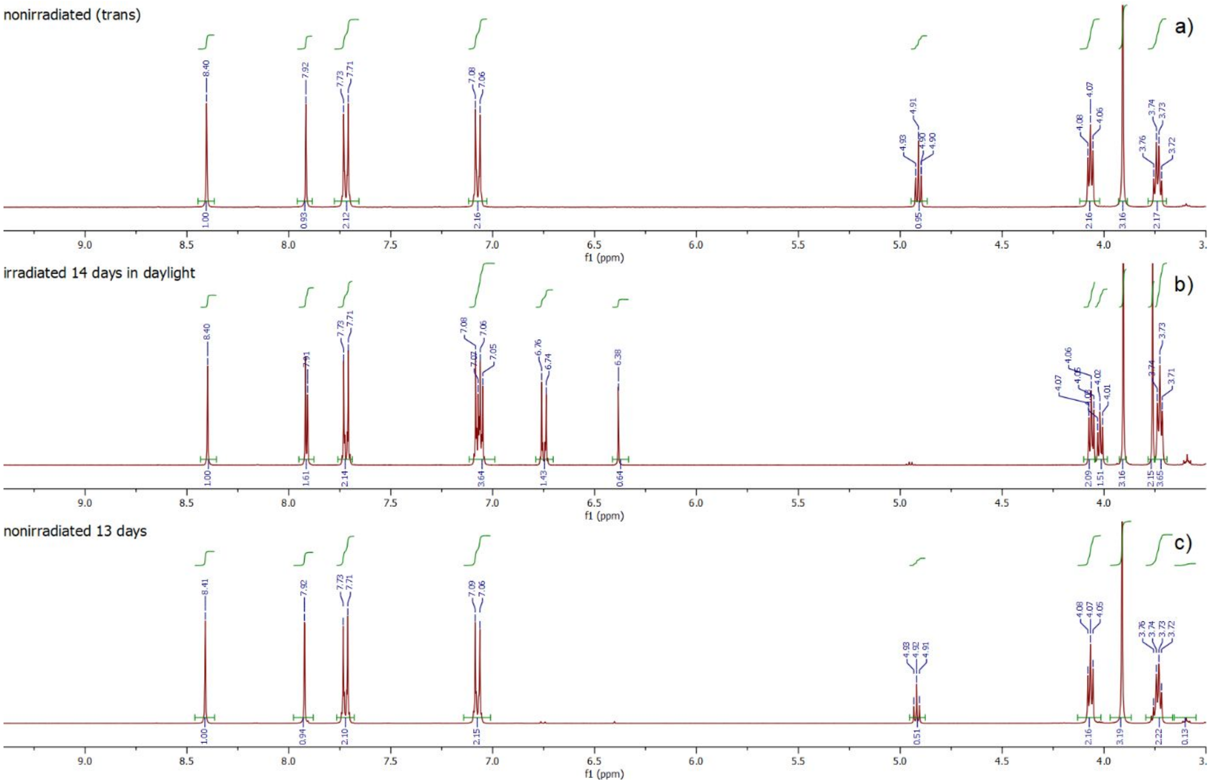

Figure S8. 1H NMR spectra of Ag(*trans*-PS1)$_2$BF$_4$ in DMSO-d6 nonirradiated measured immediately after sample preparation (a), irradiated with daylight for 14 days (b), and nonirradiated 13 days after preparation (c).

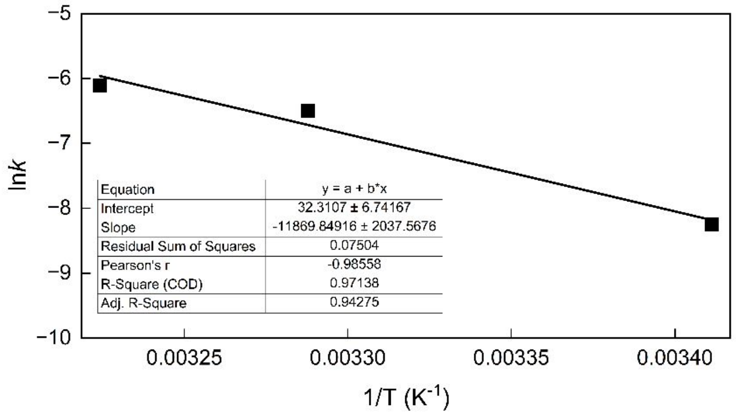

Figure S9. Dependence of $ln\ k$ on $1/T$ used to determine the value of the activation energy, E_a.

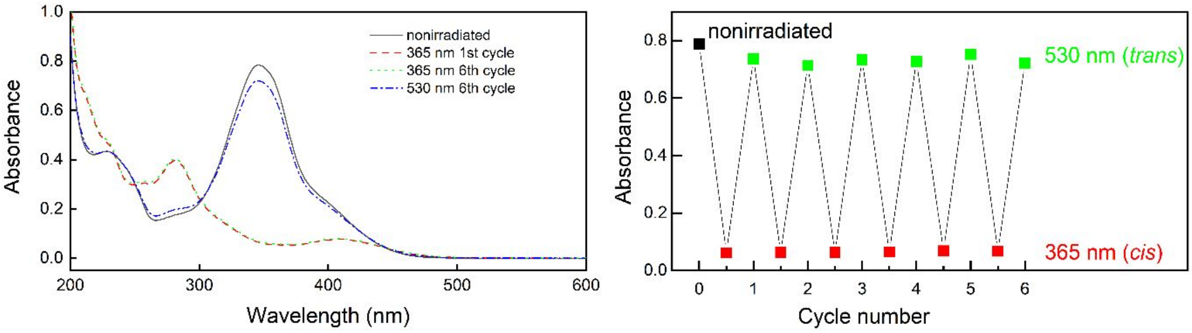

Figure S10. Absorbance at 347 nm of a 2.52 · 10^{-5} M solution of Ag(PS1)$_2$BF$_4$ nonirradiated and alternately irradiated with 365 and 530 nm light 6 times at RT. The irradiation times were 3 and 25 min, respectively, long enough to reach PSS. The power of the 365 and 530 nm lamp was 26 and 22 mW, respectively. The distance between the lamp and the cuvette: 10 cm.

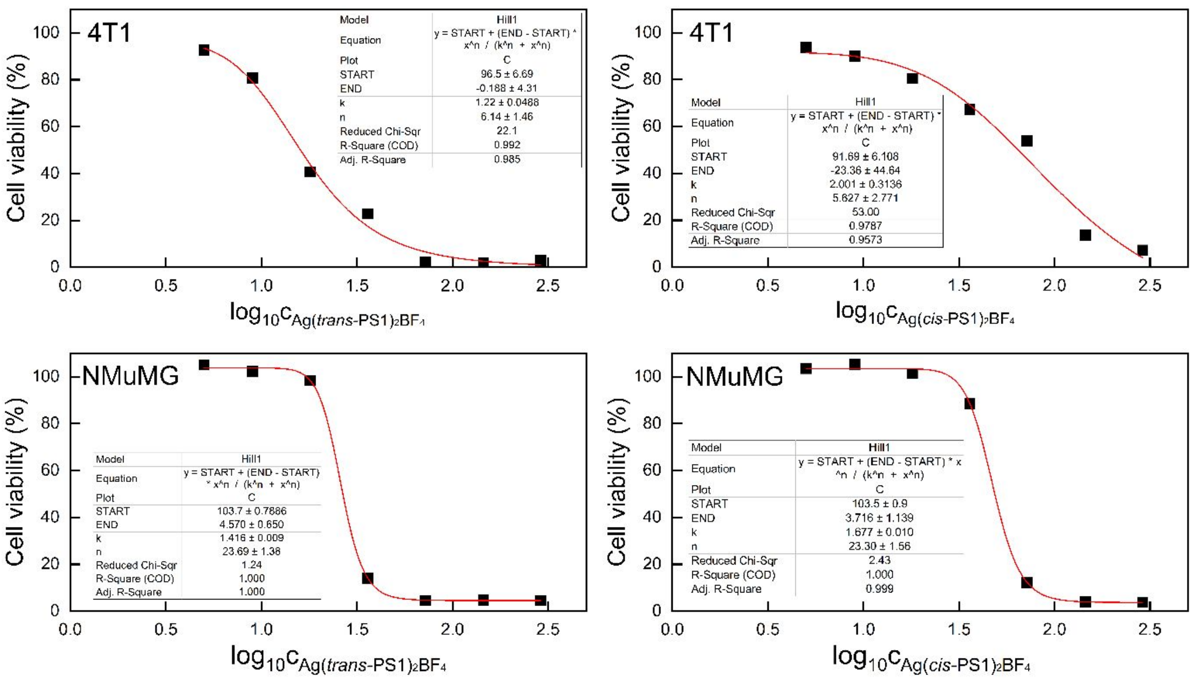

Figure S11. Cytotoxicity data of Ag(*trans*-PS1)$_2$BF$_4$ (left panels) and Ag(*cis*-PS1)$_2$BF$_4$ (right panels) in 4T1 (upper panels) and in NMuMG cells (lower panels) fitted to the Hill's equation used to determine LC50 values.
